# CORRELATES OF PHYSICAL ACTIVITY IN ADOLESCENTS OF PUBLIC SCHOOLS IN CURITIBA, PARANÁ, BRAZIL

**DOI:** 10.1590/1984-0462/2020/38/2018329

**Published:** 2020-06-12

**Authors:** Eliane Denise Araújo Bacil, Thiago Silva Piola, Michael Pereira da Silva, Rodrigo Bozza, Edmar Fantineli, Wagner de Campos

**Affiliations:** a Universidade Federal do Paraná, Curitiba, PR, Brazil.; b Universidade Estadual do Centro-Oeste, Guarapuava, PR, Brazil.; c Universidade Positivo, Curitiba, PR, Brazil.

**Keywords:** Exercise, Nutritional status, Child development, Social support, Students, Atividade física, Estado nutricional, Desenvolvimento infantil, Apoio social, Estudantes

## Abstract

**Objective:**

To verify the association of nutritional status, biological maturation, social support and self-efficacy with the physical activity level of 2,347 students of both sexes, aged between 11 and 15 years old, enrolled in state schools in the city of Curitiba, Paraná, Brazil.

**Methods:**

Anthropometric measurements of body mass, height and sitting height were collected. The assessment of biological maturation was based on the analysis of the age at peak height and sexual maturity. The physical activity level, social support from parents and friends and self-efficacy were evaluated by self-reported questionnaires. Sex/age-specific body mass index (BMI) cutoff points identified the nutritional status. Gross and adjusted binary logistic regression were used to obtain odds (OR) ratios with 95% confidence intervals (95%CI), adopting p≤0.05 as significant.

**Results:**

More than half (52.3%; n=1,227) of students were active, with boys in a higher proportion (64.1%; p≤0.01). The correlates of physical activity were: nutritional status (OR 1.25; 95%CI 1.01-1.56), early somatic maturation (OR 0.71; 95%CI 0.54-0.93), moderate (OR 1.85; 95%CI 1.50-2.30) and high social support from parents (OR 2.70; 95%CI 2.11-3.42) and high social support from friends (OR 1.78; 95%CI 1.42-2.24).

**Conclusions:**

Nutritional status, early somatic maturation, social support of parents and friends were correlates of physical activity. Overweight girls with moderate and high parental support and boys with greater social support from parents and friends were more active. Girls with early somatic maturation were less active.

## RESUMO

**Objetivo:**

Verificar a associação do estado nutricional, da maturação biológica, do apoio social e da autoeficácia com o nível de atividade física de 2.347 escolares, de ambos os sexos, com idades entre 11 e 15 anos, de escolas estaduais da cidade de Curitiba, Paraná.

**Métodos:**

Foram coletadas as medidas antropométricas de massa corporal, estatura e altura sentada. A avaliação da maturação biológica foi realizada pelas análises da idade do pico de velocidade de altura e maturação sexual. O nível de atividade física, o apoio social dos pais e dos amigos e a autoeficácia foram analisados por questionários autorreportados. O estado nutricional foi obtido por meio de pontos de corte de índice de massa corpórea (IMC) específicos para idade e sexo. Razões de chances com intervalos de confiança de 95% (IC95%) foram obtidas por meio da regressão logística binária bruta e ajustada, adotando-se p<0,05.

**Resultados:**

Pouco mais da metade (52,3%; n=1.227) dos escolares é ativa, sendo os meninos em maior proporção (64,1%; p≤0,01). Foram associados com atividade física: estado nutricional (*Odds Ratio* [OR] 1,25; IC95% 1,01-1,56), maturação somática precoce (OR 0,71; IC95% 0,54-0,93), moderado e elevado apoio social dos pais (OR 1,85; IC95% 1,50-2,30 e OR 2,70; IC95% 2,11-3,42, respectivamente) e elevado apoio social dos amigos (OR 1,78; IC95% 1,42-2,24).

**Conclusões:**

O estado nutricional, a maturação somática precoce, o apoio social dos pais e dos amigos foram correlatos da atividade física. Meninas com excesso de peso e com moderado e elevado apoio social dos pais, e meninos com maior apoio social dos pais e dos amigos foram mais ativos, enquanto meninas maturadas precocemente, menos ativas.

## INTRODUCTION

Although the benefits of physical activity (PA) are well documented in the literature, less than one in four adolescents follows recommended guidelines for PA daily.^1^ In a research carried out by Cureau et al.^2^, with Brazilian adolescents aged 12 to 17 years in municipalities with more than 100 thousand inhabitants, the prevalence of physical inactivity during leisure time was 54.3%, being higher among girls (70.7%) than boys (38%). For a better understanding of the lower levels of PA in adolescents, it is essential to consider the independent and interactive effects of the correlates that affect this behavior. The identification of such correlates will enable the implementation of behavior modification interventions in adolescence.

Biological and behavioral characteristics (biological maturation and nutritional status), as well as psychosocial characteristics (social support and self-efficacy), show greater consistency of the association with PA in adolescents. With regard to biological and behavioral characteristics, overweight individuals tend to have lower levels of PA, and insufficient PA is more prevalent among schoolchildren who present early maturation and with greater pubertal development. However, with regard to psychosocial characteristics, adolescents with more social support from parents and friends and a high perception of self-efficacy for PA have higher levels of PA.^3^ ^,^ ^4^ ^,^ ^5^ ^,^ ^6^

Studies have reported the independent association of these variables with level of PA; however, evidence on the possible influence of these variables together on PA in adolescence is still lacking.^7^ ^,^ ^8^ ^,^ ^9^ Furthermore, although the volume of literature describing the correlates of PA in adolescence is relatively large, it is highly inconsistent in terms of results and methodological quality.^10^

Studies involving correlates of PA have analytical and methodological limitations. The results and conclusions are limited by inexistent or generally low associations, studies conducted on non-representative samples, the non-consideration of external variables that influence these behaviors, and the use of instruments not validated for the population to be studied.^7^ ^,^ ^11^ ^,^ ^12^ To develop more effective interventions, the quality of this evidence base requires improvement.

In view of the need for studies with probabilistic samples, using instruments with adequate psychometric characteristics and considering external variables in the associations, this study aimed to verify the association of nutritional status, biological maturation, social support and self-efficacy with PA level in schoolchildren from Curitiba, Paraná.

## METHOD

This study is characterized as a cross-sectional correlational descriptive epidemiological survey. A stratified random sample of adolescents aged 11 to 15 years, enrolled in the day classes of elementary and high school in the state schools of Curitiba, was selected. Sample calculation took into account the following features: proportion of 50% for the prevalence of PA, 95% confidence interval (95%CI) (standard deviation [SD] = 1.96) and sample error of three percentage points, resulting in a minimum sample of 1,053 subjects. However, to correct the error related to the sample selection process, a design effect of 1.5 was added, which resulted in a minimum sample of 1,579 students.

To this estimate, an additional 30% of individuals (474 students) were added to minimize losses related to refusal to participate in the study, failure to deliver the informed consent form signed by parents or guardians and the free and informed consent term until the day of collection, and the presence of one of the following exclusion criteria: incorrect questionnaire filling, missing or incomplete data; lack of anthropometric data and biological maturation; gestation; physical disability; and withdrawal from participation in the study. Given these criteria, the total sample was estimated at 2,052 students, 1,026 boys and 1,026 girls.

The total sample size was 2,697 students. Of these, 114 adolescents were outside the age group of interest, two were physically disabled and one was pregnant. In addition to these losses, 36 adolescents did not complete all items in the questionnaire and 108 completed them incorrectly. Cases of refusal to participate in data collection were rare. However, there were 63 cases of loss due to failure to deliver the informed consent form signed by parents/guardians and 26 withdrew from participating in the study. Therefore, the final sample was composed of 2,347 adolescents.

The calculation of the statistical power of this final sample was carried out later on in GPower 3.1.7, for the 95%CI (α = 0.05), and found that the sample has the power to detect significant Odds Ratio (OR) for PA equal to or greater than 1.14 with 92% power.

The sampling process was carried out in three stages. Initially, all state schools were listed and stratified according to each of the ten administrative regions in the city of Curitiba. A school was drawn in each of the ten administrative regions of the city, which guaranteed the representativeness of the geographic areas of the city in the sample, and a simple random selection was made of two classes each year, according to the number of students (separated by sex) required for a given administrative region.

Data collection was carried out from March to May 2016 by a trained team from the Center for Studies in Physical Activity and Health (CEAFS in the Portuguese acronym) of *Universidade Federal do Paraná* (UFPR). A pilot study was previously carried out to train evaluators about the procedures, aiming to improve the reliability of data collection. The evaluators completed and applied the questionnaires to colleagues, as well as were measured and performed anthropometric and sexual maturation measures on colleagues.

Before data collection, an authorization was requested from the State Education Secretariat (SEE) and also from the students’ parents and the students themselves - through the informed consent forms - to participate in the research. The study was approved by the Research Ethics Committee at UFPR (Opinion No. 722,529; CAAE 30350514.3.0000.0102), according to Resolution No. 466/2012 of the National Health Council (CNS).

The adolescents filled out a structured questionnaire in the classroom containing: information on sociodemographic features, PA, social support and self-efficacy. The anthropometric assessment was carried out in the school’s Physical Education room. Subsequently, the maturation stage was assessed in another reserved room, making sure that the researcher was of the same sex as the adolescent being evaluated.

The sociodemographic characteristics collected in the study were: work (yes and no), housing (living with father and mother, father or mother and others), type of residence (house/two-floor house; apartment/other) and education of father and mother (<8 years of study and ≥8 years of study).

The following moderating and controlling variables were used: gender, age and economic class. The students were grouped into two age groups (11 to 12 years old, and 13 to 15 years old) due to the breadth of the age group and the different perception of students in both groups. The determination of the economic class was based on the Brazil Economic Classification Criterion (CCEB),^13^ which accounts for the number of items in each student’s household. The students were instructed to answer whether each item on the list and respective quantities were present in their households, the level of education of the head of the family or the person responsible for supporting them, and the presence of a public service (running water and paved street). The students were classified in economic classes A/B (superior) and C/D/E (medium/low).

For the analysis of nutritional status, measures of body weight and height were collected initially, following the procedures described by Alvarez and Pavan.^14^ The classification of nutritional status was determined by body mass index (BMI) adjusted for age and sex, as proposed by Cole et al.^15^ The students were classified as eutrophic and overweight (overweight + obese).

Biological maturation was evaluated by analyzing somatic maturation and sexual maturation. Somatic maturation was assessed by the age of peak height velocity (PHV)^16^ using anthropometric data, including height, seated height, estimated leg length, body mass and chronological age. Specific equations for males (equation 1) and females (equation 2) were developed to estimate the maturity offset or the number of years of PHV.^16^ The classification of somatic maturation was performed by age of PVH: boys (early: <13.07; time: 13.07 to 14.63; and late:> 14.63) and girls (early: <11.61; time: 11.61 to 12.81; and late:> 12.81).

Sexual maturation was determined by Tanner’s stages.^17^ This method was determined by comparative self-assessment with boards illustrating the appearance of pubic hair in both girls and boys. Sexual maturation was classified into: stage 1 (prepubertal), stage 2 (pubertal) and stage 3 (postpubertal).

Social support and self-efficacy for PA were assessed using a four-point Likert scale.^18^ The social support scale for PA contains 12 items, covering different types of social support for PA that students can receive from parents or friends. The social support questionnaire is divided into two sessions: parents and friends. In each of the sessions, the six types of social support are specified: stimuli, practice together, transport, watching, comments and talking. Among friends, the variable transport has been replaced by inviting. Based on a typical or normal week, students reported the frequency (never, rarely, often or always) with which parents and friends encourage them to engage in PA. The self-efficacy scale for PA has ten items: 1 (strongly disagree), 2 (disagree), 3 (agree) and 4 (strongly agree).^18^ Subsequently, these scores were added up. The scales of social support and self-efficacy were classified in tertiles: 1st tertile (low), 2nd tertile (moderate) and 3rd tertile (high). The scales of social support and self-efficacy showed reproducibility and satisfactory validity.^19^

The PA questionnaire analyzed in the present study is an adaptation of the Self-Administered Physical Activity Checklist.^20^ The Physical Activity Questionnaire for Adolescents proposed by Farias Junior et al.^21^ and adapted from Sallis et al.^20^ consists of a list of 24 Moderate to vigorous PA (>3METS), with the possibility for the student to add two. When completing the questionnaire, the students informed the frequency (days/week) and duration (hours and minutes per day) of the PA in the previous week. Of the 24 questions, one refers to locomotion to PA: “walking as a means of transportation (walking to school, work, a friend’s house) [considering the round-trip time].” The question regarding PA in leisure time was removed from the analyses. In determining the level of PA, the sum of the time spent in each PA by respective practice frequencies greater than 420 minutes/week was considered “sufficiently active”, and less time than that as “insufficiently active”.^22^ This questionnaire showed good reproducibility and adequate validity.^23^

When analyzing data, the normality of data set was initially verified by the Kolmogorov-Smirnov test and by histograms (asymmetry and kurtosis coefficients). The description of categorical variables was obtained by the distribution of absolute and relative frequency (total and stratified by sex). The chi-square test was used for comparisons between genders.

Binary logistic regression was used to verify the association between correlates and PA by creating three prediction models. The association of biological and behavioral variables (model 1) and psychosocial variables (model 2) with PA was verified. Adjusted analyses (model 3) with PA were used. These were performed in the Statistical Package for the Social Sciences (SPSS) version 21.0, with significance level set at p<0.05.

## RESULTS

Table 1 shows the sociodemographic characteristics of the students. 2,347 adolescents participated in the study, a little more than half of whom were males. Most participants were aged 13 to 15 years, belonged to the upper economic class (A/B), lived with their father and mother in a house/two-floor house, and whose father and mother had more than eight years of study.

**Table 1:** Sociodemographic variables, total and stratified by sex, of school adolescents aged 11 to 15 years from Curitiba, Paraná (n = 2,347).

	Total		Male		Female		chi-square	p-value
	n	%	n	%	n	%		
Sex								
Male	1,204	51.3	-	-	-	-		
Female	1,143	48.7	-	-	-	-		
Age group								
11-12 years	933	39.8	440	36.5	493	43.1	10.35	**0.01**
13-15 years	1,414	60.2	764	63.5	650	56.9		
Work								
Yes	152	6.5	107	8.9	45	3.9	22.91	**0.01**
No	2,195	93.5	1.097	91.1	1.098	96.1		
Household								
With father and mother	1,446	61.6	741	61.5	705	61.7	0.01	0.98
With father or mother	737	31.4	380	31.6	357	31.2		
Other	164	7.0	83	6.9	81	7.1		
Type of household								
House/two-floor house	2,056	87.6	1.050	87.2	1.006	88.0	0.28	0.60
Apartment/other	291	12.4	154	12.8	137	12.0		
Father´s schooling								
<8 years of study	570	30.9	288	30.5	282	31.4	0.13	0.71
≥8 years of study	1,272	69.1	656	69.5	616	68.6		
Mother´s schooling								
<8 years of study	564	29.0	278	28.3	286	29.7	0.41	0.52
≥8 years of study	1,384	71.0	706	71.7	678	70.3		
Economic class								
A/B (high)	1,523	64.9	824	68.4	699	61.2	13.34	**0.01**
C/D/E (medium/low)	824	35.1	380	31.6	444	38.8		

Table 2 shows information on nutritional status, somatic maturation and sexual maturation, social support from parents and friends, and the students’ self-efficacy. Among them, 26% of adolescents were overweight, and most were mature in time (72.2%) and pubertal (79.7%).

**Table 2.** Nutritional status, somatic and sexual maturation, social support from parents and friends and self-efficacy, total and stratified by sex, of school adolescents aged 11 to 15 in Curitiba, Paraná (n = 2,347).

	Total		Male		Female		chi-square	p-value
	n	%	n	%	n	%		
Nutritional status								
Eutrophic	1,737	74.0	882	73.3	855	74.8	0.65	0.42
Overweight	610	26.0	322	26.7	288	25.2		
Somatic maturation								
Maturation on time	1,654	72.2	885	75.3	769	68.8	10.82	**0.01**
Early maturation	338	14.7	155	13.2	183	16.4		
Late maturation	300	13.1	135	11.5	165	14.8		
Sexual maturation								
Prepubertal	71	3.1	34	2.9	37	3.3	88.45	**0.01**
Pubertal	1,832	79.7	853	72.5	979	87.3		
Postpubertal	395	17.2	289	24.6	106	9.4		
Social support from parents								
Low (1st tertile)	859	36.6	383	31.8	476	41.6	29.19	**0.01**
Moderate (2nd tertile)	811	34.6	426	35.4	385	33.7		
High (3rd tertile)	677	28.8	395	32.8	282	24.7		
Social support from friends								
Low (1st tertile)	833	35.5	347	28.8	486	42.5	83.10	**0.01**
Moderate (2nd tertile)	674	28.7	320	26.6	354	31.0		
High (3rd tertile)	840	35.8	537	44.6	303	26.5		
Self-efficacy								
Low (1st tertile)	795	33.9	429	35.6	366	32.0	8.64	**0.01**
Moderate (2nd tertile)	772	32.9	412	34.2	360	31.5		
High (3rd tertile)	780	33.2	363	30.1	417	36.5		
Physical activity								
<420 minutes	1,120	47.7	432	35.9	688	60.2	137.95	**0.01**
>420 minutes	1,227	52.3	772	64.1	455	39.8		

Table 3 shows the associations between biological and behavioral characteristics (nutritional status, somatic maturation, and sexual maturation) and psychosocial characteristics (social support from parents and friends and self-efficacy) with schoolchildren’s PA. Nutritional status, somatic maturation and social support from parents and friends were associated with PA. Overweight students with support from parents and friends for PA practice were more likely to be active than eutrophic students who did not receive support from parents and friends to engage in PA. Regarding the association of somatic maturation with PA, students who were classified as having early maturation were less active (OR 0.71; 95%CI 0.54-0.93).

**Table 3:** Association of biological and behavioral characteristics (nutritional status, somatic and sexual maturation) and psychosocial (social support from parents and friends and self-efficacy) with physical activity of school adolescents aged 11 to 15 in Curitiba, Paraná.

	Active		Crude analysis	Adjusted analysis*
	n	%	OR (95%CI)	OR (95%CI)
Biological and behavioral characteristics				
Nutritional status				
Eutrophic	901	50.42	1.0	1.0
Overweight	347	55.70	**1.25 (1.02-1.53)**	**1.25 (1.01-1.56)**
Somatic maturation				
Maturation on time	907	52.82	1.0	1.0
Early maturation	172	50.89	0.81 (0.63-1.04)	**0.71 (0.54-0.93)**
Late maturation	141	47.00	0.81 (0.63-1.03)	1.05 (0.79-1.40)
Sexual maturation				
Pubertal	955	50.69	1.0	1.0
Prepubertal	40	56.34	1.26 (0.77-2.04)	1.09 (0.64-1.85)
Postpubertal	225	55.42	**1.26 (1.01-1.58)**	1.13 (0.87-1.45)
Psychosocial features				
Social support from parents				
Low (1st tertile)	318	35.69	1.0	1.0
Moderate (2nd tertile)	455	55.02	**1.95 (1.59-2.39)**	**1.85 (1.50-2.30)**
High (3rd tertile)	475	68.64	**3.02 (2.40-3.79)**	**2.70 (2.11-3.42)**
Social support from friends				
Low (1st tertile)	352	40.88	1.0	1.0
Moderate (2nd tertile)	333	47.84	1.14 (0.92-1.41)	1.12 (0.89-1.40)
High (3rd tertile)	563	66.00	**1.99 (1.61-2.47)**	**1.78 (1.42-2.24)**
Selfefficacy				
Low (1st tertile)	431	52.95	1.0	1.0
Moderate (2nd tertile)	399	50.19	0.84 (0.68-1.03)	0.91 (0.72-1.13)
High (3rd tertile)	418	52.18	0.87 (0.71-1.08)	1.01 (0.80-1.25)

OR: Odds Ratio; 95%CI: 95% confidence interval; *adjusted for all independent variables and for control variables: sex, age and economic class.

Table 4 shows the associations between biological and behavioral characteristics (nutritional status, somatic maturation and sexual maturation) and psychosocial characteristics (social support from parents and friends and self-efficacy) with schoolchildren’s PA, stratified by sex. Boys who received social support from parents and friends were more likely to be active than students with low social support. Overweight girls and receiving support from parents to practice PA were more likely to be active. However, early matured girls tend to be less active.

**Table 4.** Association of biological and behavioral characteristics (nutritional status, somatic and sexual maturation) and psychosocial (social support from parents and friends, and self-efficacy) with physical activity, stratified by sex, of school adolescents aged 11 to 15 years in Curitiba, Paraná.

	Male (n=1.204)				Female (n=1.143)			
	Active		Crude analysis	Adjusted analysis*	Active		Crude analysis	Adjusted analysis*
	n	%	OR 95%CI	OR 95%CI	n	%	OR 95%CI	OR 95%CI
Biological and behavioral characteristics								
Nutritional status								
Eutrophic	562	63.7	1.0	1.0	324	37.9	1.0	1.0
Overweight	210	65.2	1.15 0.86-1.55	1.11 0.81-1.52	131	45.5	**1.33** **1.01-1.78**	**1.43** **1.05-1.94**
Somatic maturation								
Maturation on time	573	64.7	1.0	1.0	313	40.7	1.0	1.0
Early maturation	94	60.6	0.78 0.53-1.14	0.74 0.49-1.11	78	42.6	0.99 0.70-1.39	**0.66** **0.45-0.98**
Late maturation	84	62.2	0.94 0.64-1.38	1.12 0.74-1.71	57	34.5	0.79 0.56-1.13	0.96 0.62-1.49
Sexual maturation								
Pubertal	543	63.7	1.0	1.0	393	40.1	1.0	1.0
Prepubertal	22	64.7	1.09 0.52-2.30	1.00 0.45-2.22	18	48.6	1.47 0.76-2.85	1.13 0.56-2.28
Postpubertal	186	64.4	1.05 0.80-1.40	1.19 0.87-1.63	37	34.9	0.81 0.53-1.25	0.98 0.62-1.54
Psychosocial features								
Social support from parents								
Low (1st tertile)	191	49.9	1.0	1.0	122	25.6	1.0	1.0
Moderate (2nd tertile)	274	64.3	**1.58** **1.18-2.12**	**1.57** **1.16-2.12**	175	45.5	**2.38** **1.77-3.20**	**2.18** **1.60-2.97**
High (3rd tertile)	307	77.7	**2.60** **1.87-3.63**	**2.47** **1.73-3.50**	158	56.0	**3.42** **2.46-4.75**	**2.90** **2.06-4.09**
Social support from friends								
Low (1st tertile)	176	50.7	1.0	1.0	167	34.4	1.0	1.0
Moderate (2nd tertile)	193	60.3	1.34 0.97-1.84	1.36 0.98 - 1.88	134	37.9	0.91 0.67-1.23	0.93 0.68-1.27
High (3rd tertile)	403	75.0	**2.17** **1.59-2.95**	**2.31** **1.67-3.19**	154	50.8	1.35 0.98-1.85	1.34 0.97-1.87
Self-efficacy								
Low (1st tertile)	281	65.5	1.0	1.0	142	38.8	1.0	1.0
Moderate (2nd tertile)	252	61.2	0.78 0.58-1.05	0.83 0.61-1.13	139	38.6	0.90 0.66-1.23	1.00 0.72-1.39
High (3rd tertile)	239	65.8	0.93 0.68-1.26	0.90 0.66-1.25	174	41.7	0.99 0.74-1.34	1.13 0.83-1.56

OR: Odds Ratio; 95%CI: 95% confidence interval; *adjusted for all independent variables and for control variables: age and economic class.

## DISCUSSION

The results showed that just over half of the students are active, with boys in a greater proportion. The differences between genders in engagement in PA can reflect different social roles imposed by society that influence the differences of interests between boys and girls. Culturally, boys prefer to play sports and participate in sports competitions, which usually involves vigorous PA, in contrast to girls, more inclined to perform activities with little energy expenditure, which can lead them to be less active naturally.^24^

As for the correlates of PA, overweight students tend to be more active. However, this trend is more evident in girls. It is speculated that the girls’ constant concern with the current aesthetic standards imposed by society favors the demand for PA practice to reduce body weight.

Early matured students tend to practice less PA, and this trend is more evident among girls. These results are in accordance with the literature. Studies indicate that PA tends to decrease as age advances, and one of the explanations may be biological age.^25^ Thus, adolescents become less physically active as they progress towards maturity. Difference in the timing of growth spurt may be relevant to this decline in PA. In a systematic review carried out by Bacil,^26^ early matured girls were less active.

Early matured girls may decrease their interest in the practice of PA due to the physical changes typical of adolescence, such as increased fat deposits, greater breast development and enlarged hips. They also report more negative experiences in PA, for example, injuries and lack of ability due to little experience in different types of PA; they are given greater restrictions and limits to leave home and meet friends; as well as, in this phase, the increase in the obligations of daily tasks, work at home and/or the transition from school to work can favor the performance of more sedentary activities.^27^ Conversely, the physical changes that occur in boys, such as gain in height, body mass, a higher proportion of lean mass and broadening of the shoulders, are beneficial for participation in PA, as these result in a physical contribution more suitable for success in many forms of PA, particularly those that emphasize speed, power and strength.^26^

School adolescents who had greater social support from parents and friends were more likely to be active. In a systematic review carried out by Mendonça et al.,^28^ social support was positively associated with the levels of PA of adolescents in both cross-sectional and longitudinal studies. Those who received greater social support from parents and friends showed higher levels of PA. Social support for PA provided by parents occurs through logistical support, such as providing transportation or covering transportation costs to the site where they practice activities, participating in activities with adolescents, as well as support and encouragement to adhere to leisure PA. However, the social support provided by friends is linked to the sharing of values, norms, tastes and preferences, which directly influences the adolescent’s choice and adherence to PA.^7^ As boys have greater freedom to find friends outside the school environment, whether on the street, parks and sports courts, or simply playing on the street, they are more susceptible to practicing PA and being influenced by friends.^29^

Self-efficacy was not associated with gender-independent PA. In both boys and girls, the perception of confidence in their ability to perform and maintain active behavior did not influence the level of PA of the students in this study. This behavior is different from that observed in the study by Souza et al.,^30^ in which the association between self-efficacy and PA was stronger for girls compared to boys. What can explain, in part, these differences is the instrument used and the different age range between the studies. The period between 11 and 15 years is a period of many doubts, crises and ambivalences among adolescents, which can cause low self-esteem and inferiority in relation to other colleagues. These factors favor the decrease in self-efficacy for PA.

Regional characteristics may have influenced the results of this study. Curitiba, capital of Paraná, is a city known for its urban planning and for its various green areas, such as parks and squares, which may favor the demand for the practice of PA by overweight girls, to reduce body weight. In addition, it is considered the coldest capital in Brazil, which can influence the importance of social support from parents and friends related to PA.

Thus, the importance of regional surveys for the production of knowledge is shown, indicating the general health status of school adolescents. Behavioral characteristics are studied in order to assess the health condition of school-age individuals, favoring the creation of public policies. According to this logic, studying PA practice and the factors that influence it is essential to improve the health conditions and quality of life of the studied population.

The present study has strengths that deserve to be highlighted. The research analyzed the relationship between biological and behavioral variables (nutritional status and biological maturation) and psychosocial variables (social support and self-efficacy) with PA in a representative sample of school adolescents from Curitiba, Paraná. Another strength was the adequate sample size for the analysis of association between variables, in addition to the use of previously tested instruments that were shown to have acceptable levels of reproducibility and validity.

This study also had some limitations. One of them was the use of self-reported measures to assess PA, since it depends a lot on the subjects’ understanding of the variables being evaluated.

The results of the present study showed that most students are active, boys in greater proportion. Correlates of PA were considered: nutritional status, biological maturation, social support from parents and friends. Overweight girls with moderate and high social support from parents and boys with greater social support from parents and friends tend to be more active, and early matured girls, less active.

The evidence presented in this study may support future interventions for the promotion of PA, as they contribute to findings that reinforce the consideration of biological and behavioral (nutritional status and biological maturation) and psychosocial (social support and self-efficacy) aspects as important variables in targeted studies better understanding of PA practice behavior.

Thus, the results of the present study reinforce the need to intervene in the correlates of PA for behavioral change in adolescents. Intervention programs to promote PA must consider nutritional status, biological maturation and social support from parents and friends for behavior change. More research is needed to incorporate prospective longitudinal study designs, objective methods to assess PA, as well as the analysis of mediating variables that better explain these relationships.
